# Correction: The structural and luminescence properties of plexcitonic structures based on Ag_2_S/l-Cys quantum dots and Au nanorods

**DOI:** 10.1039/d2ra90021a

**Published:** 2022-03-17

**Authors:** Irina G. Grevtseva, Oleg V. Ovchinnikov, Mikhail S. Smirnov, Aleksey S. Perepelitsa, Tamara A. Chevychelova, Violetta N. Derepko, Anna V. Osadchenko, Alexandr S. Selyukov

**Affiliations:** Voronezh State University, Department of Optics and Spectroscopy Voronezh Russia Grevtseva_IG@inbox.ru; Voronezh State University of Engineering Technologies Voronezh Russia; Bauman Moscow State Technical University Moscow Russia; P.N. Lebedev Physical Institute of the Russian Academy of Sciences Moscow Russia; Moscow Institute of Physics and Technology Dolgoprudnyi Moscow Oblast Russia

## Abstract

Correction for ‘The structural and luminescence properties of plexcitonic structures based on Ag_2_S/l-Cys quantum dots and Au nanorods’ by Irina G. Grevtseva *et al.*, *RSC Adv.*, 2022, **12**, 6525–6532, DOI: 10.1039/D1RA08806H.

The authors regret the omission of a funding acknowledgement in the original article. This acknowledgement is given below.

This study was supported by the Ministry of Science and Higher Education of the Russian Federation under Agreement N 075-15-2021-1351 as part of the structural analysis of colloidal Ag_2_S/l-Cys QDs and Au NRs.

The Royal Society of Chemistry apologises for these errors and any consequent inconvenience to authors and readers.

## Supplementary Material

